# mTOR-Mediated Regulation of Immune Responses in Cancer and Tumor Microenvironment

**DOI:** 10.3389/fimmu.2021.774103

**Published:** 2022-02-18

**Authors:** Sahar Mafi, Behzad Mansoori, Shahram Taeb, Hossein Sadeghi, Reza Abbasi, William C. Cho, Davoud Rostamzadeh

**Affiliations:** ^1^ Department of Clinical Biochemistry, Yasuj University of Medical Sciences, Yasuj, Iran; ^2^ Medicinal Plants Research Center, Yasuj University of Medical Sciences, Yasuj, Iran; ^3^ The Wistar Institute, Molecular & Cellular Oncogenesis Program, Philadelphia, PA, United States; ^4^ Department of Radiology, School of Paramedical Sciences, Guilan University of Medical Sciences, Rasht, Iran; ^5^ Medical Biotechnology Research Center, School of Paramedical Sciences, Guilan University of Medical Sciences, Rasht, Iran; ^6^ Department of Clinical Oncology, Queen Elizabeth Hospital, Hong Kong, Hong Kong SAR, China

**Keywords:** cancer, mTOR, T cell, Tumor microenvironment, PI3K/Akt/mTOR signaling pathway, immune response

## Abstract

The mechanistic/mammalian target of rapamycin (mTOR) is a downstream mediator in the phosphatidylinositol 3-kinase (PI3K)/Akt signaling pathways, which plays a pivotal role in regulating numerous cellular functions including cell growth, proliferation, survival, and metabolism by integrating a variety of extracellular and intracellular signals in the tumor microenvironment (TME). Dysregulation of the mTOR pathway is frequently reported in many types of human tumors, and targeting the PI3K/Akt/mTOR signaling pathway has been considered an attractive potential therapeutic target in cancer. The PI3K/Akt/mTOR signaling transduction pathway is important not only in the development and progression of cancers but also for its critical regulatory role in the tumor microenvironment. Immunologically, mTOR is emerging as a key regulator of immune responses. The mTOR signaling pathway plays an essential regulatory role in the differentiation and function of both innate and adaptive immune cells. Considering the central role of mTOR in metabolic and translational reprogramming, it can affect tumor-associated immune cells to undergo phenotypic and functional reprogramming in TME. The mTOR-mediated inflammatory response can also promote the recruitment of immune cells to TME, resulting in exerting the anti-tumor functions or promoting cancer cell growth, progression, and metastasis. Thus, deregulated mTOR signaling in cancer can modulate the TME, thereby affecting the tumor immune microenvironment. Here, we review the current knowledge regarding the crucial role of the PI3K/Akt/mTOR pathway in controlling and shaping the immune responses in TME.

## Introduction

The mammalian target of rapamycin (mTOR; now officially known as the mechanistic target of rapamycin) is a ubiquitous serine/threonine-specific protein kinase, plays a critical role in regulating numerous cellular functions, including cell growth, proliferation, survival, protein synthesis, ribosome biogenesis, autophagy, and metabolism (1, 2). mTOR functions within two functionally and structurally distinct multi-component kinase complexes called mTOR complex 1 (mTORC1) and mTOR complex 2 (mTORC2) that act as the central nodes of the phosphoinositide 3-kinase (PI3K)/Akt downstream signaling pathway (3). The activity of PI3K/Akt/mTOR pathway is frequently dysregulated in majority of human tumors and has a crucial role during tumorigenesis and cancer development (4–6). Thus, targeting the PI3K/Akt/mTOR signaling pathway would be an attractive potential therapeutic target in cancer (7).

The tumor microenvironment (TME) contains malignant cells and nonmalignant cells such as endothelial cells, cancer-associated fibroblasts (CAFs), and several kinds of tumor-infiltrating immune/inflammatory cells, as well as a variety of soluble factors (cytokines and growth factors) released from cell subpopulations and plays pivotal roles in facilitating tumorigenesis, promoting tumor progression and immune evasion (8). TME is highly enriched in the immune cell populations and reciprocal signaling between immune cells and cancer cells can reduce the anti-cancer activity of endogenous tumor-infiltrating immune cells and facilitate immune evasion (9, 10). Studies have shown that the immune infiltration of tumors are closely associated with tumor proliferation, angiogenesis, invasion, and metastasis (11). Along with the critical role of the mTOR in cancer, recent studies have established an essential regulatory role of the mTOR in differentiation, activation, and functional properties of immune cells in which mTOR functions to coordinate and shape immune effector responses (12, 13). Therefore, dysregulation of this network potentially affects immune cells effector function and influences the tumor immune microenvironment (TIME) landscape in human cancers (10). As the core regulator of metabolic and translational reprogramming, mTOR is mainly involved in the central tumor immune microenvironment, affecting tumor-associated immune cells to undergo phenotypic and functional reprogramming in TME. Indeed, mTOR regulates immune responses by regulating the expression of inflammatory mediators, such as interleukin (IL)-12, IL-10, transforming growth factor (TGF-β), and tumor necrosis factor (TNF), as well as immune checkpoint receptors cytotoxic T-lymphocyte protein 4 (CTLA-4) and programmed death 1 (PD-1) (12, 14). Further, a recent study has revealed that mTOR gene expression is markedly correlated with various immune cells and immunoinhibitors in patients with clear cell renal cell carcinoma (ccRCC) (15). The mTOR-mediated inflammatory response also promotes immune cells recruitment to TME by inflammatory mediators, resulting in exerting the anti-tumor functions or augments tumor growth, progression, and metastatic capacities of cancer cells (14).

This review focuses on the current knowledge regarding the key role of the mTOR signaling pathway in controlling and shaping the immune responses in TME.

## mTORC1 Signaling Pathway

**Figure 1:**
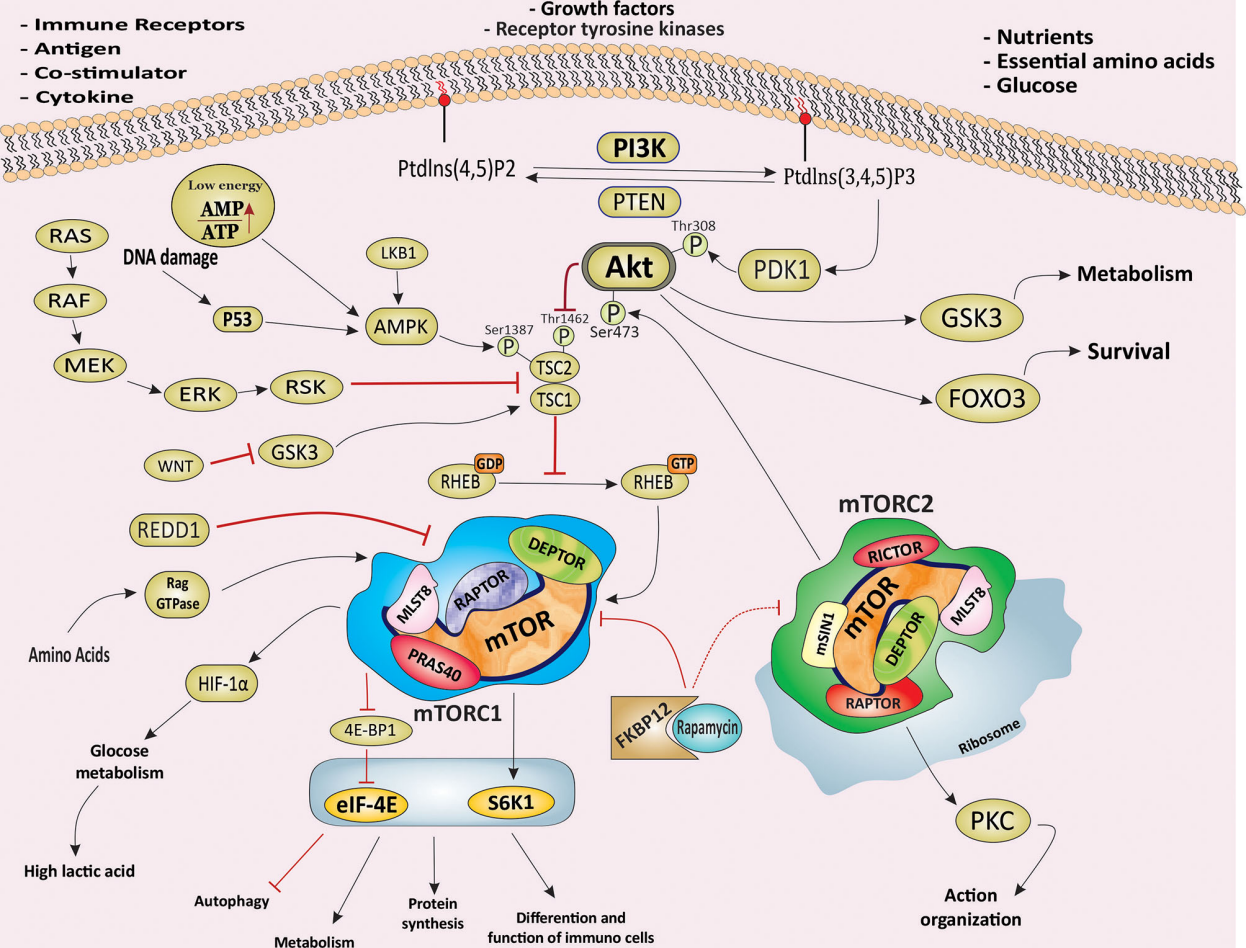
The PI3K/Akt/mTOR signaling pathway and signaling pathway of mTORC1 and mTORC2. Schematic illustration of the molecular components and signaling events related to the PI3K/Akt/mTOR signaling pathway and its main downstream effectors. Schematic showing the main molecular components and signals sensed by mTORC1 and mTORC2 and the processes they regulate to control main cellular events, including growth, protein synthesis, metabolism, survival and proliferation. mTOR, mechanistic target of rapamycin; PI3K, phosphoinositide 3 kinase; mTORC1, mTOR complex 1; mTORC2, mTOR complex 2; RAPTOR, regulatory-associated protein of mTOR; RICTOR, rapamycin-insensitive companion of mTOR; mSIN1, mammalian stress-activated protein kinase interacting protein; eIF4E, eukaryotic initiation factor 4E; 4EBP1, eIF4E -binding protein 1; S6K1, S6 kinase 1; HIF-1α, Hypoxia Inducible Factor 1α; PKC, Protein kinase C; AMPK, AMP activated protein kinase; SGK1, glucocorticoid regulated kinase 1; FKBP12, FK506 Binding Protein 12; LKB1, liver kinase B1; DEPTOR, DEP-containing mTOR interacting protein; mLST8, mammalian lethal with Sec13 protein; RAPTOR, regulatory-associated protein of mTOR; PRAS40, proline-rich AKT substrate 40 kDa; mSin1, mammalian stress-activated protein kinase (SAPK)-interacting protein 1; Rheb, RAs homologue enriched in brain; TSC, tuberous sclerosis complex; GSK3, Glycogen synthase kinase-3; PDK1, Phosphoinositide-dependent Kinase 1; FOXO3, Forkhead box family transcription factors 3; PTEN, phosphatase and tensin homologue; REDD1, regulated in development and DNA damage responses 1.

mTOR is an atypical serine/threonine-specific protein kinase that belongs to the phosphatidylinositol-3 kinase-related kinases (PIKK) family (16). mTOR functions as a downstream effector of the PI3K/Akt signaling pathway in two distinct sets of intracellular complexes, mTORC1 and mTORC2 (3). Both complexes share three conserved subunits: mTOR, the catalytic subunit, DEP-containing mTOR interacting protein (DEPTOR), and mammalian lethal with SEC13 protein 8/G protein β subunit-like (mLST8/GβL). In addition to the same subunits, the regulatory-associated protein of mTOR (RAPTOR) and proline-rich Akt substrate 40 kDa (PRAS40) are unique subunits for mTORC1 complex, whereas rapamycin-insensitive companion of mTOR (RICTOR), protein observed with Rictor (PROTOR) and mammalian stress-activated protein kinase (SAPK)-interacting protein 1 (mSIN1) are unique components for mTORC2 complex **(** **Figure 1** **)** (16). The mTOR pathway senses a variety of intracellular and extracellular signals in the form of growth factors, cytokines, nutrients, and energy levels in the form of ATP, cellular stress, and inflammation. The signaling inputs downstream of these diverse signals are predominantly delivered to the PI3K/Akt pathway that eventually activates mTOR **(** **Figure 1** **)** (1, 12). The phosphatase and tensin homologue (PTEN), a well-characterized tumor suppressor, negatively regulates PI3K/Akt/mTOR signaling pathway. In numerous cancer types, perturbations of PTEN regulation or PTEN loss-of-function mutations consequently result in upregulation of PI3K/Akt/mTOR signaling which contributes, to tumorigenesis (17). The tuberous sclerosis complex (TSC) is a key upstream negative regulator of mTORC1 kinase activity, which exists in a heterodimer that comprises TSC1 and TSC2 (also called harmatin and tuburin, respectively). TSC2 functions as a GTPase-activating protein (GAP) which inhibits the activity of RAS homologue enriched in brain (Rheb) as an essential activator of mTORC1 (18). Activation of RAS–MAPK (mitogen-activated protein kinase) and PI3K/Akt signaling induces the inhibitory phosphorylation of TSC2 and results in dissociation of The TSC1/TSC2 complex, which in turn leads to activation of mTORC1 signaling via Rheb. Thus, suppression of TSC allows the GTP-bound Rheb to bind and activate mTORC1 **(** **Figure 1** **)** (19, 20). Under normal conditions, activation of mTORC1 leads to promoting cap-dependent translation initiation and protein synthesis mandatory for cell growth and proliferation, mainly *via* direct phosphorylation of ribosomal protein S6 kinases (S6Ks) and eukaryotic translation initiation factor (eIF4E)-binding proteins (4E-BPs) (20, 21). Phosphorylated S6K1 promotes mRNA translation and cell growth by phosphorylation of ribosomal S6 and eIF-4B. The mTOR‐dependent phosphorylation of 4E‐BP1 potentially disrupts its binding to eIF4E, which also stimulates translation (12, 21). mTOR signaling also regulates and activates numerous transcription factors, such as c-MYC, hypoxia-inducible factor 1-α (HIF1-α), STAT3, transcription factor EB (TFEB), peroxisome proliferator-activated receptor-γ (PPARγ), PPARα, and sterol regulatory element-binding proteins (SREBPs) (22). Also, mTORC1 mediated suppression of autophagy, mainly through the phosphorylation of the serine/threonine kinase Unc-51-like kinase 1 (ULK1), essential core factors of the autophagy, along with the phosphorylation of S6Ks (S6K1 and S6K2) and 4E-BP1, are essential for cell growth (23). In addition, phosphorylation of PRAS40^Thr246^ by PKB/Akt, and PRAS40^Ser183^ and PRAS40^Ser221^ by mTORC1, an inhibitory component of mTORC1, leads to the dissociation of the PRAS40 from mTORC1, and its binding to 14-3-3 proteins results in indirect activation of mTORC1, independent of TSC1/2 (24).

Rapamycin (Sirolimus) is a macrolide compound produced by a strain of *Streptomyces hygroscopicus*, and first discovered in soil samples collected from Rapa Nui (Easter Island) with potent and selective antifungal activity (25). Subsequently, rapamycin and its analogues, Everolimus (RAD001), Temsirolimus (TRM-986), Ridaforolimus (AP23573, MK-8669), and Zotarolimus (ABT-578), were found to have both immunosuppressant and anti-tumor potentials, and they emerged as a promising class of novel anti-tumor agents (5, 26). Rapamycin binds to a cytoplasmic receptor FK506 Binding Protein 12 (FKBP12), and this complex then interacts with the FKBP12–rapamycin‐binding (FRB) domain of mTOR and rapidly inhibits mTORC1 downstream signaling **(** **Figure 1** **)** (12). Activation of AMP-activated protein kinase (AMPK) by sensing low cellular energy status and nutrient starvation in the form of intracellular AMP levels, ischemia, as well as under hypoxic conditions leads to an increase in the TSC1/TSC2-mediated inhibition of Rheb-mTORC1 pathway *via* phosphorylation of TSC2 (27). In addition, glycogen synthase kinase 3 (GSK3)-mediated phosphorylation of TSC2 promotes the inhibition of the mTOR pathway. Wnt signaling inhibits GSK3 resulting in activation of the mTOR pathway **(** **Figure 1** **)** (28). In brief, mTORC1 activation induces cap-dependent translation *via* activation of at least two independent targets, S6K1 and 4EBP1/eIF4E, resulting in increases in mammalian cell size and proliferation which are two common features of cancer (29, 30).

## mTORC2 Signaling Pathway

While mTORC1 is highly sensitive to rapamycin-FKBP12 complex, mTORC2 is relatively insensitive to long-term rapamycin treatment and is characterized by its sensitivity to prolonged rapamycin treatment. Rapamycin-mediated inhibition of mTORC2 is thought to be through suppression of mTORC2 assembly, likely as a result of abrogating the binding of rapamycin/FKBP12 to newly synthesized mTOR to RICTOR (31). Whereas the upstream signals derived from a wide range of extracellular and intracellular causes such as amino acids, growth factors, and cellular functions of mTORC1 are well characterized, little information is known regarding the upstream signals and the biological significance of mTORC2. However, several findings provide new insights into the fundamental role of mTORC2 in the regulation of various biological processes, including cell growth, survival, metabolism, cell migration, proliferation, and cytoskeleton organization and identification of its other physiological and cellular functions are also an open line of investigation (16, 32). mTORC2 is a critical regulator of Akt, a crucial serine/threonine kinase in cellular processes and frequently deregulated in many types of human cancer **(** **Figure 1** **)** (32, 33). A positive feedback loop between Akt and mTORC2 is necessary for the full activation of Akt. Phosphoinositide-dependent Kinase 1 (PDK1)-dependent phosphorylation of Akt at tyrosine 308 promotes the Akt kinase activity. Subsequently, activated Akt augments mTORC2 kinase activity, resulting in phosphorylation of Akt at serine 473 by mTORC2, which is necessary for full activation of Akt (34). Activated Akt also phosphorylates TSC2, resulting in blockage of TSC2 and TSC1 combination. For downstream effectors, mTORC2 mediated phosphorylation of protein kinase C (PKC) family members, small GTPase RAs homologue (RHO), and serum and glucocorticoid−regulated kinase 1 (SGK1) is a key step in critical cellular processes **(** **Figure 1** **)** (32).

mTORC2 activates N-myc downstream-regulated gene 1 protein (NDRG1) and forkhead box family transcription factors (FOXO), which enhance the cell survival in the response of normal and cellular stresses such as oxidative stress, DNA damage, and nutrient deprivation as well as cancer cells in response to hypoxic stress (35, 36). Moreover, mTORC2-mediated activation of PKC family members is involved in regulating cytoskeleton reorganization and cell motility, migration, and invasion involved in tumorigenesis (37, 38). It has been demonstrated that PI3K-mediated activation of mTORC2 resulting in enhancing mTORC2-ribosome binding, suggesting that ribosomes activate mTORC2 directly. mTORC2-ribosome interaction subsequently facilitated Akt signaling pathway activation in cancer cells **(** **Figure 1** **)** (39). Further understanding of mTORC2 dysregulation and its physiological functions holds enormous potential to bring regarding that mTORC2 could serve as a novel and amenable therapeutic targets for human disorders, including cancer.

## The Critical Role of mTOR Signaling Pathway in Cancer

**Figure 2:**
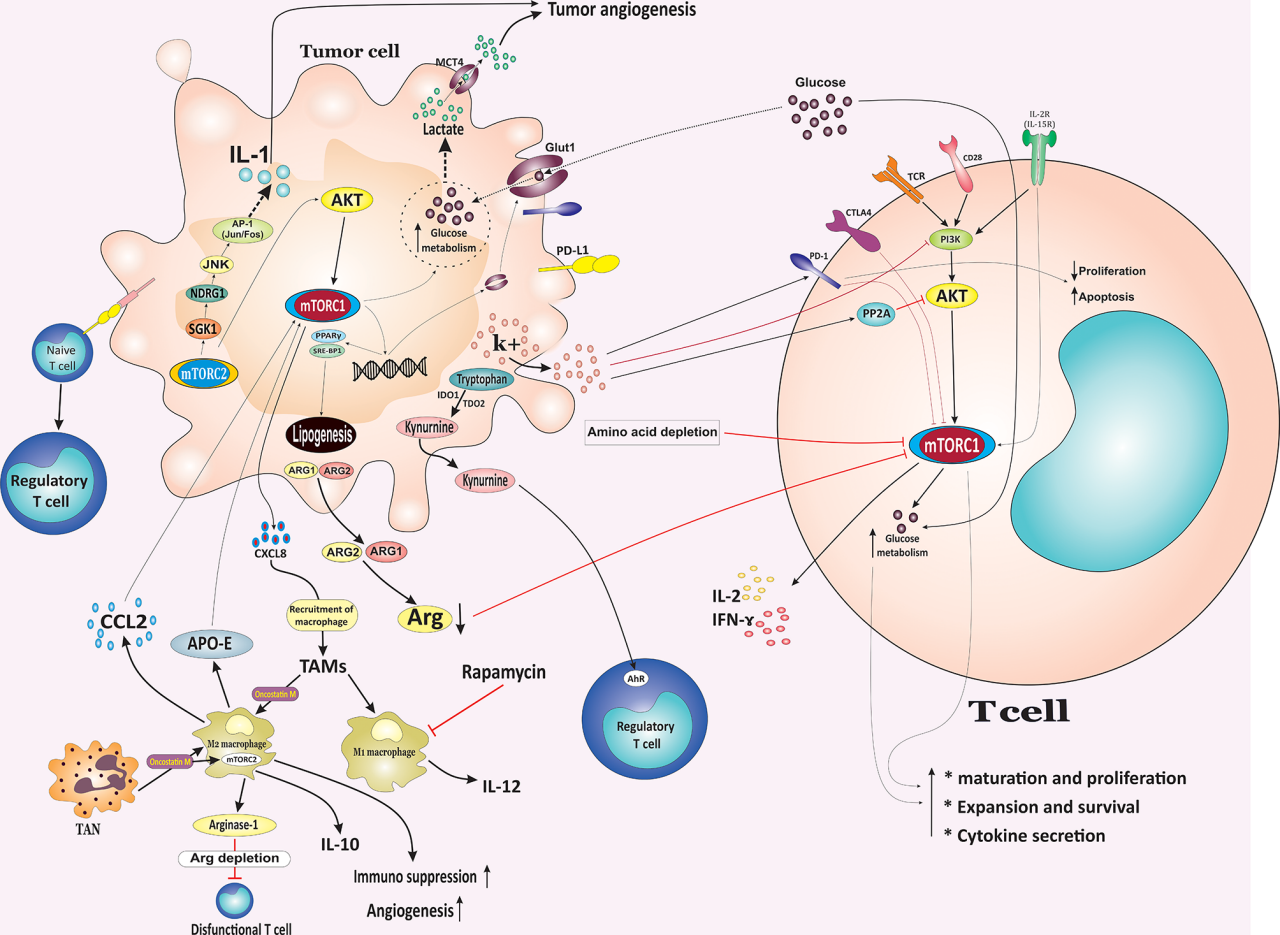
Metabolic competition between tumor cells and T cells in the tumor microenvironment. The nutrient interplay between malignant and nonmalignant cells, especially immune cells, can potentially influence cancer cells growth, survival, and function. Tumors might inhibit immunity through nutrient consumption and simultaneously produce metabolites to inhibit T cell function, resulting in tumor escape from the immune system. In TME, tumor cells outcompete T cells for glucose, leading to sustained mTOR signaling and glycolysis in the tumor cells. Additionally, increased glycolysis by tumor cells leads to the release of lactate *via* MCT4, which enhances immunosuppression and angiogenesis. In contrast to PD-1 and CTLA-4, which inhibit the mTOR-mediated upregulation of glycolysis, the co-stimulation of the CD28 signaling pathway and signaling mediated *via* cytokines such as IL-2 and IL-15 activates PI3K/Akt/mTOR pathway to promote the switch of the T cell metabolism to glycolysis. Tumor cells also affect T cells *via* releasing high-[K+]e, which induced inhibition of TCR-mediated Akt and mTOR phosphorylation and induced the upregulation of PD-1. Cancer cells also evade immune responses by releasing cytosolic ARG1 or mitochondrial ARG2 results in diminished levels of arginine and reduced mTOR activity and inhibition of T cells in the TME. Additionally, upregulation of IDO1 and TDO2 by cancer cells leads to the tryptophan degradation into kynurenine which supports the Treg phenotype in an AhR repentant manner. Overexpression of mTORC2 target SGK1 mediates tumor growth and invasion in different cancer types. Overexpression of the SGK1 target, NDRG1, is suggested to stimulate IL-1 expression and promote tumor angiogenesis through JNK/AP-1 activation. Both mTORC1 and mTORC2 signaling induce lipogenesis and fatty acid uptake through promoting SREBP1 and PPARγ. CCL2 secreted by TAMs render cancer cells resistance through activating the PI3K/Akt/mTOR pathway. TME, tumor microenvironment; mTOR, mammalian target of rapamycin; MCT4, monocarboxylate transporter 4; ARG1, arginase 1; IDO1, indoleamine-2,3-dioxygenase 1; TDO2, tryptophan-2,3-dioxygenase 2; AhR, aryl hydrocarbon receptor; Treg, regulatory T cell; SREBP1, sterol regulatory element-binding protein 1; PPARγ, peroxisome proliferator-activated receptor-γ; CCL2, CC‐chemokine ligand 2; TAMs, tumor associated macrophages; PD-1, Programmed cell death protein 1; CTLA4, Cytotoxic T-Lymphocyte Associated Protein 4; NDRG1, N-myc downstream-regulated gene 1 protein; MCT4, monocarboxylate transporter 4.

Given the crucial role of the mTOR pathway in different fundamental cellular processes, several lines of evidence have identified that the dysregulation of the PI3k/Akt/mTOR signaling pathway closely contributes to the various human pathological conditions including, tumor initiation and progression, maintenance, and metastasis **(** **Table 1** **)** (4–6). Aberrant hyperactivation of the mTOR pathway in cancer mainly results from different levels of mechanisms and its signal cascade **(** **Table 1** **)**. First, mutations in the mTOR gene lead to constitutive activation of mTOR, which has been reported in a few human cancers. In a recent study by Grabiner et al., comprehensive cancer-associated mTOR mutations identified thirty-three mutations using publicly available tumor genome sequencing datasets after generating a comprehensive catalogue of mTOR pathway mutations in cancer. The detected mutations clustered in six distinct regions in the C-terminal half of mTOR, and these were accompanied by different cancer types, with one cluster particularly prominent in kidney cancer. Interestingly, these mutations were contributed to the mTOR pathway hyperactivation by inhibiting the interaction of mTOR and its endogenous inhibitor mTOR inhibitor DEPTOR, but did not affect mTOR complex assembly (55). Second, genetic aberrations in the specific components of both mTORC1 and mTORC2 in the non-small cell lung cancer (NSCLC) and breast cancer, respectively, which was associated with poor prognosis and short disease-free survival (41, 56). Third, hyperactivation of the mTOR pathway can arise from mutations in upstream elements including, tumor suppressors and oncogenes, which in physiological conditions render it activation or suppression, respectively. Multiple mutations in PI3K signaling pathway members, a bona fide upstream signal pathway of both mTORC1 and mTORC2, have been frequently described in human cancers. Activating mutations and amplification in PIK3CA, the gene encoding the p110α catalytic subunit of PI3K have been reported to frequently mutated in different types of human cancer such as prostate (29%), breast (27%), endometrium (23%), colon (15%), upper aerodigestive tract, etc (10%) (46). As a hallmark of proliferating cancer cells, metabolic reprogramming is a critical strategy of cancer cells to alter their metabolism and promote their biological capabilities to ensure their growth, survival, and rapid proliferation. As a master regulator of cellular metabolism, mTOR-mediated upregulation of protein synthesis at the level of S6K1 and 4E-BP1/eIF-4E plays a crucial role in this scenario (57). Glutamine is necessary as a nitrogen and carbon donor for the major biosynthetic pathways such as the synthesis of amino acids, lipids, and nucleotides, which are used by cancer cells to replenish the tricarboxylic acid (TCA) cycle metabolites *via* a process known as anaplerosis (6). mTORC1 promotes glutamine synthesis *via* positive regulation of glutamate dehydrogenase (GDH) (58) and by repressing sirtuin 4 (SIRT4), a GDH inhibitor (59). mTOR facilitates cancer cell growth and proliferation by promoting glucose metabolism. Aerobic glycolysis has been regarded as a hallmark of cancer cells, and glutamine provides the main source of carbon and nitrogen for facilitating anabolic processes and supporting cell growth (60). mTOR signaling promotes reprogramming of glucose metabolism by increasing the expression of transporter 1 (Glut1) and glucose uptake (61), as well as by inducing the expression of the transcription factors c-MYC and HIF1-α (62), which play a crucial role in the induction of several other glycolytic enzymes such as phosphoglucoisomerase (PGI), phosphofructokinase (PFK) and enolase (ENO) **(** **Figure 2** **)** (6).

**Table 1:** The role of PI3K/Akt/mTOR signaling pathway components in cancer.

Genes	Consequences of the alteration	Refs
***Akt***	Amplification and overexpression of Akt have been reported in many cancer types.	(40)
***Rictor***	*Rictor* is amplified in a subset of human cancers, such as lung and breast cancers, and is associated with cancer progression and therapeutic resistance. There is an association between *RICTOR* amplification and sensitivities to mTOR1/2 inhibitors in non-small cell lung cancer cells.	(41–43)
***PI3K***	PI3K mutations and activity is associated with cell transformation, cancer initiation, and tumor progression. High PI3K activity has been reported in many human cancers such as ovarian, gastrointestinal, pancreatic, breast, and prostate.	(40, 44–46)
***PTEN***	PTEN is a tumor-suppressor gene that acts as a major regulator of the PI3K/Akt/mTOR signaling pathway. Impaired PTEN function results in tumor initiation and progression and has been described in a large proportion of human cancers.	(46)
***Rheb***	Rheb is the major activator of mTORC1 and frequently overexpressed in human carcinomas and induces multistage carcinogenesis through induction of multiple oncogenic mechanisms.	(47)
***TSC1/TSC2***	Loss of function mutations in *TSC1* and *TSC2* genes results in constitutive mTOR activation and tumor progression.	(48)
***S6K1***	S6K1 is considered a critical downstream target for mTOR and is abnormally activated in a wide range of human cancers. Overexpression of S6K1 is associated with poor prognosis in many cancer types such as esophageal squamous cell carcinoma (ESCC), non-small cell lung cancer (NSCLC), breast cancer, and nasopharyngeal carcinoma (NPC).	(49, 50)
***4EBP1***	Overexpression of 4EBP1 has shown to be associated with tumorigenesis and poor prognosis in many human cancers, such as breast, head and neck, colon, and prostate.	(51–54)

RICTOR; rapamycin-insensitive companion of mTOR; 4EBP1; eIF4E -binding protein 1; S6K1; S6 kinase 1, The following abbreviations are used; mTOR; mechanistic target of rapamycin; PI3K; phosphoinositide 3 kinase; mTORC1; mTOR complex 1; mTORC2; mTOR complex 2; TSC; tuberous sclerosis complex; PTEN; phosphatase and tensin homologue; Rheb; RAS homologue enriched in brain.

Amplified *de novo* lipogenesis is considered a hallmark of proliferating cancer cells (63). Both mTORC1 and mTORC2 signaling induce lipogenesis and fatty acid uptake, which is required for cell growth and proliferation, through promoting SREBP1 and PPARγ, two critical transcription factors which facilitates the expression of several enzymes involved in lipid and cholesterol homeostasis, including ATP citrate lyase (ACLY), Acetyl-Coenzyme A carboxylase 1 (ACC1), fatty acid synthase (FASN) as well as the fatty acid transporter CD36 **(** **Figure 2** **)** (16, 64, 65).

Although the fundamental role of the mTORC1 signaling pathway has been widely studied in various types of cancer, several recent insights into the function of the mTORC2 pathway uncovered the critical roles for mTORC2 in different cancer types such as prostate, breast, lung cancer, glioblastoma (GBM), pancreatic cancer, and T-cell acute lymphoblastic leukemia (T-ALL) **(** **Table 1** **)** (41, 43, 66–68). Specific ablation of the mTORC2 component, *RICTOR*, significantly delays pancreatic tumorigenesis. Interestingly, combined inhibition of mTORC1/2 and PI3K significantly prolonged survival in late-stage tumor *in-vivo*. Suggesting that targeting mTORC2 as a potential therapeutic strategy for the clinical intervention of pancreatic cancer (68). Overexpression of RICTOR has been detected in 74.0% of gastric cancers (69) and was associated with tumor progression, lymph node metastasis, and poor prognosis in patients with gastric cancer (69). Targeting of mTORC2 either by kinase inhibitors or RICTOR knockdown induces apoptosis of breast cancer cells and suppresses cell migration and metastasis (70, 71). Additionally, RICTOR deficiency results in a substantial decrease in pAktSer^473^ level and significantly reduces the proliferation of colorectal cancer cells and tumor growth (72). Glucose-induced RICTOR acetylation promotes mTORC2 activation, and driving therapeutic resistance to inhibitors of the PI3K pathway result in promoting epidermal growth factor receptor vIII (EGFRvIII)-dependent signaling in glioblastoma cells (73). RICTOR upregulation was found to be contributed to the hyperactivation of Akt, aggressive breast cancers, and decreased overall survival. Additionally, ablation of RICTOR/mTORC2 signaling subsequent RICTOR knockdown or treatment with mTORC1/2 dual kinase reduced Akt-mediated tumor cell survival and promoted lapatinib-mediated cell killing in HER2^+^ breast cancer cells, a dual HER2/EGFR tyrosine kinase inhibitor (41). Metabolic reprogramming-mediated by mTORC2 has been shown to promote tumorigenesis, where it augments tumor growth by providing lipids necessary for growth and energy production. In liver-specific PTEN^−/−^ and Tsc1^−/−^ mouse models, oncogenic activation of mTORC2 resulting in activation of SREBP1 which promotes *de novo* fatty acid and lipid synthesis such as sphingolipids, glycerophospholipids, and cardiolipins to increase mitochondrial respiration (74). These results highlighs the importance of mTORC2 signaling in cancer, and mTOR inhibition serves as a promising therapeutic strategy for the clinical intervention of cancer.

## The PI3K/Akt/mTOR Signaling Network and Tumor Microenvironment

The TME is complex and composed of diverse cell types and non-cellular components such as numerous growth factors, cytokines, chemokines, and other tumor-promoting molecules released or created by the extracellular matrix (75). Nonmalignant cell components of TME include CAFs, adipocytes, vascular endothelial cells, and different immune cell types such as regulatory T cells (Treg), tumor-associated macrophages (TAM), myeloid-derived suppressor cells (MDSCs), B lymphocytes, dendritic cells (DCs), natural killer (NK) cells and natural killer T (NKT) cells, (1, 76). The nonmalignant cells component of TME exhibit a dynamic and often tumor-promoting role at all stages of carcinogenesis (76). Emerging evidence indicates that the crucial regulatory role of the PI3K/Akt/mTOR axis in differentiation, activation, homeostasis, and effector functional properties of immune cells to coordinate and shape the innate and adaptive immunity (12, 77). PI3K/Akt/mTOR axis can sense and integrate inputs from a variety of environmental signals in the context of TME to regulate the immune cell trafficking, polarization, and their functional properties to promote tumor progression and metastasis. Likewise, activation of the PI3K/Akt/mTORC1 pathway is critical for the development and metabolic reprogramming of effector CD4^+^ and CD8^+^ T cells (78, 79). Both mTORC1 and mTORC2 mainly function as crucial signaling nodes that receive and integrate multiple upstream input signals from T cell receptor (TCR) (known as signal 1), several costimulatory molecules (known as signal 2), and different cytokine exposure (known as signal 3) to coordinate the downstream signaling programs for regulating immune receptor signaling pathways, transcriptional and metabolic programming and migratory activity. The signal transduction pathway mediated by mTOR ultimately determines the T cell homeostatic and dictates immune cell fate decisions in effector, memory, and Tregs cells (13, 80). Rheb-deficient T cells were unable to generate Th1 and Th17 responses *in vitro* and *in vivo* and failed to induce classical experimental autoimmune encephalomyelitis (EAE). However, they sustained their ability to become Th2 cells. Alternatively, specific elimination of mTORC2 signaling by selective conditional deletion of the RICTOR in T cells blocked selective Th2 cells development *in vitro* and *in vivo* but preserved their ability to differentiate into Th1 and Th17 cells. Additionally, selective deletion of both mTORC1 and mTORC2 signaling were necessary for the generation of immunosuppressive Treg cells in the absence of exogenous transforming growth factor (TGF)-β (78).

Metabolic reprogramming-mediated by mTOR complexes implicates in orchestrating the interaction of TME components, particularly immune cells and neoplastic cells, suggesting the crucial role of PI3K/Akt/mTOR in the determination of tumor development, progression as well as drug resistance. Thus, with regard to the many actions in which mTOR is involved, the inhibition of PI3K/Akt/mTOR holds enormous potential to bring about novel therapeutic targets or strategies for reducing cancer cells proliferation, migration, invasion, and survival and enhancing the efficacy of the tumor immunosurveillance through both the downregulation of the immunosuppressive pathways and the activation of anti-tumor immunity in combination with agents able to negate immune suppression and boost T cell immunity. However, recent studies have demonstrated that the mTOR blockade has surprising immunostimulatory effects by enhancing the generation of memory precursor effector cells that survive and differentiate into long-lived CD8^+^ memory cells, allowing better clearance of tumor cells (81). Consequently, the pharmacologic or genetic targeting of key components of this signaling pathway is a potential therapeutic target for clinical intervention patients with a range of different cancers.

## CD4^+^ Subsets and CD8^+^ T Lymphocytes and Tumor Cells in TME: In Competition for Nutrition

As was mentioned above, malignant cells undergo metabolic reprogramming characterized by enhancing glucose uptake and aerobic glycolysis, glutamine uptake and glutaminolysis, oxidative phosphorylation (OXPHOS), and lipid metabolism. These metabolic alterations support critical metabolites and energy for rapid malignant proliferation, growth, invasion, metastasis in a nutrient fluctuating environment (6). Similar to cancer cells, immune cells also undergo metabolic reprogramming during development, activation, and effector or memory differentiation, resulting in distinct functional fates (82). T cells are divided into several distinct subtypes and can destroy target tumor cells directly or indirectly by synthesizing and releasing various biological molecules. In addition, each subset of T cells exhibits distinct unique metabolic demands for biological energy and biosynthesis and signaling pathways that contribute to its fate and function (82). Naïve T cells undergo extensive changes in their metabolic properties during proliferation, differentiation, and capacity to differentiate into distinct effector subtypes as well as their effector function. Naïve T cells mostly rely on lower metabolic demand and have a catabolic metabolism by which they use glucose, fatty acids, and amino acids for ATP generation through the TCA cycle and OXPHOS. Upon antigen stimulation, T cells significantly alter their metabolism to support these increased synthetic demands, and the cells transition into anabolic metabolism mediated by glycolysis and glutaminolysis to obtain energy for cell growth, proliferation, differentiation, and cytokine secretion (80).

In the context of heterogeneous TME, the nutrient interplay between malignant and nonmalignant cells, especially immune cells, can potentially influence tumor cells growth, survival, and function. Tumors might inhibit immunity through nutrient consumption and simultaneously produce metabolites to inhibit T cell function, resulting in tumor escape from the immune system. Within the TME, tumor cells outcompete T cells for glucose, leading to sustained mTOR signaling and glycolysis in the tumor cells and, consequently, tumor progression. Conversely, decreased glucose concentrations following enhanced glucose consumption by tumor cells result in downregulation of mTOR activity in antitumor immune cells, glycolysis, and chemokine secretion (83). In turn, the downregulation of mTOR activity in T cells impairs their metabolic reprogramming and function and facilitates tumor immune escape. Additionally, increased glycolysis by tumor cells leads to the release of lactate *via* monocarboxylate transporter 4 (MCT4), which promotes immunosuppression and angiogenesis **(** **Figure 2** **)** (84).

Glucose deprivation or reduction in TME following consumption by cancer cells metabolically restricts aerobic glycolysis in tumor-infiltrating T cells, resulting in T cell dysfunction and impairing T cell-mediated immunosurveillance and enhancing immunosuppressive properties of tumor-infiltrating lymphocytes (TILs) (83, 85). In addition, glycolysis is obligatory for T cell maturation, expansion, and effector function during an immune response (86). Nutrient competition distinctly can affect TILs activity, and antigen-specific T cell effector function can be affected by tumor cell numbers and glucose concentrations *in vivo*. In this regard, TILs showed a reduction in mTOR activity and interferon (IFN)-g production. Therefore, mTOR activity may directly or indirectly reflect the nutritional and functional status of immune cells and cancer cells **(** **Figure 2** **)** (83). Ligations of immune checkpoint receptors CTLA-4 and PD-1 have been revealed to suppress the PI3K/Akt/mTOR signaling pathway resulting in diminished IL-2 production, Bcl-xL expression, glucose uptake, and glycolytic rate (87). PD-1 is expressed by activated T lymphocytes and is a crucial immune checkpoint receptor that mediates immunosuppression upon binding to its ligand PD-1 ligand-1 (PD-L1) expressed by tumor cells. They are considered to be the central mediator of immunosuppression in the tumor immune TME. Loss of PTEN expression and subsequently constitutive PI3K/mTOR activation contributed to the upregulating cell surface PD-L1 expression in triple-negative breast cancer (TNBC). Interestingly, treatment of breast cancer cells with either the Akt inhibitor or the mTOR inhibitor was associated with a markedly decrease in PD-L1 cell surface expression **(** **Figure 2** **)**. Furthermore, elevated levels of PD-L1 expression following PTEN knockdown results in decreased proliferation and increased apoptosis in activated T cells and raise the possibility that targeting the PI3K/mTOR pathway as a therapeutic strategy may augment adaptive immune responses against cancer (88). Targeting the PD-L1/PD-1 pathway has consistently shown significant and promising therapeutic efficacy in patients with advanced cancers (89). However, PD-L1 is well known to inhibit T cells *via* PD-1, it is now elucidated that it serves additional biological advantages for tumors. Chang et al. showed that PD-L1 on the tumor cell surface sustains Akt/mTOR signaling, which in turn promotes glycolysis by enhancing the glycolysis enzymes (83). Therefore, PD-L1 blockade therapy reduced mTOR activity and mTOR–mediated upregulation of glycolysis in tumor cells, which, in turn allowing more available glucose within the extracellular milieu of the tumor and restores TILs glycolytic capacity and, as a result, their effector function (83). Hence, anti-PD1 treatment not only blocks inhibitory receptors but also can suppress tumor growth by directly regulating tumor cell metabolism *via* modulating mTOR activity and reducing the glucose exhaustion in TME, which promotes CD8^+^ cytotoxic T lymphocyte (CTL) functions, suggesting a very effective and promising advancement for therapies targeting both tumor immunity and TME.

In contrast to PD-1 and CTLA-4 which, inhibit the mTOR-mediated upregulation of glycolysis, the co-stimulation of CD28 signaling pathway and signaling mediated *via* cytokines such as IL-2 and IL-15 activates PI3K/Akt/mTOR pathway to promote the switch the T cell metabolism to glycolysis **(** **Figure 2** **)** (90, 91). Therefore, preventing the metabolic activity of tumor cells by modulating the PI3K/mTOR pathway may have the potential enormous to enhance glucose availability to T cells, thereby enhancing the antitumor activity of T cells.

Tumor cells can also influence the TME by releasing extracellular signals which, derive suppression of T cell function in the TME. For example, dying or necrotic tumor cells release high-[K^+^]e, which induces the inhibition of TCR-mediated Akt and mTOR phosphorylation and upregulates inhibitory protein PD-1 which, resulting in profound effects on tumor-resident T cell metabolic pathway and induces T cell suppression (92). The elevated K^+^ concentration leads to more K^+^ entering into the T cells through a pump or leak channels resulting in an increase in intracellular K^+^ levels. In this milieu, elevated K^+^ drives hypophosphorylation of the Akt/mTOR pathway and suppression of T cells in a PP2A-dependent manner **(** **Figure 2** **)** (93). PTEN loss and PI3K/Akt pathway activation in melanoma are associated with resistance to T cell-mediated tumor killing, reduced T cell infiltration at tumor sites, likely due to increased secretion of immunosuppressive cytokines by cancer cells, and resistance to immune checkpoint inhibitors (94). The emerging evidence highlights that sensing of amino acids by the mTOR pathway in immune cells is crucial for their proliferation, metabolism, and activation (95). T cell activation is associated with the rapid uptake of amino acids such as glutamine and leucine that is essential for appropriate metabolic reprogramming (96). Indeed, an influx of branched-chain amino acids (BCAAs) such as leucine and glutamine regulate a broad range of immune cell functions through modulating mTORC1 activation. Sensing of amino acids such as arginine, glutamine, and leucine regulate the recruitment and localization of mTORC1 from the cytoplasm to the lysosomal surface *via* the RAS-related GTP-binding proteins (RAGs), where enhances mTORC1 activity by bringing the complex in contact with Rheb, which strongly stimulates the kinase activity of mTORC1 **(** **Figure 1** **)** (97). The Rag complex, specially RagD, is required for CD8^+^ T cell antitumor immunity, and RagD deficiency induces a dysfunctional phenotype in CD8^+^ TILs. Amino acids promote the RagD-mediated translocation of mTORC1 to lysosomes, which render the maximal mTORC1 activity in CD8^+^ TILs. Tumor cells reduce T cell access to leucine and therefore impair leucine-driven mTORC1 activation, which eventually impairs the CD8^+^ TIL antitumor immunity (98).

Cancer cells activity impacts immune cells by depleting specific amino acids in the tumor milieu and creating an immunosuppressive TME. For example, the release of cytosolic arginase 1 (ARG1) or mitochondrial arginase 2 (ARG2) results in diminished levels of arginine, reduced mTOR activity, and inhibition of T cells in the TME (99). Upregulation of indoleamine-2,3-dioxygenase 1 (IDO1) and tryptophan-2,3-dioxygenase 2 (TDO2) by cancer cells result in the tryptophan degradation into kynurenine. While tryptophan deprivation suppresses proliferation and promotes apoptosis in T cells in the TME, secreted kynurenine supports Treg cells phenotype in an aryl hydrocarbon receptor (AhR) repentant manner, which further suppresses immune responses (100). T cell suppression may result from a reduction in mTORC1 following a reduction in intracellular amino acid levels. Additionally, overexpression of mTORC2 target SGK1 is correlates with tumor growth and invasion in various cancers (101). In gastric tumors, overexpression of the SGK1 target, NDRG1, is suggested to stimulate IL-1 expression and induce tumor angiogenesis through JNK/AP-1 activation. NDRG1 is considered as a metastasis suppressor in many cancer types (102). Additionally, NDRG1 overexpression is associated with enhanced upregulation of angiogenic CXC chemokines, vascular endothelial growth factor A (VEGF-A), matrix metalloproteinase (MMP)-1, and cell invasion, which supports angiogenesis and malignant progression **(** **Figure 2** **)** (103).

Taken together, these data suggest that enhanced PI3K/Akt/mTOR signaling in cancer cells may affect T cells fate, and thereby influencing tumorigenesis and malignant progression.

## mTOR Regulates Treg/Th17 Balance in the Tumor Microenvironment

Treg cells are an immunosuppressive subset of CD4^+^ T lymphocytes, characterized by the expression master transcription factor forkhead box protein P3 (Foxp3). Foxp3^+^ Tregs play an indispensable role in maintaining self‐tolerance and cancer immune evasion through their role as a suppressor of the effector T cells. High numbers of tumor-infiltrating Foxp3^+^ Tregs and decreased ratios of tumor-infiltrating CD8^+^ T cells in the tumor infiltrate are correlated with promoting tumor development and progression in several tumor types (104, 105). Under normally activating conditions, the reduction in mTOR signaling following mTOR inhibition is associated with Treg expansion. Both pharmacological and genetic disruption of the mTOR signaling induce expansion of the expansion of Tregs by promoting the expression of Foxp3 (106). Conversely, the PI3K/Akt/mTORC1 signaling pathway promotes Th17 cell differentiation. mTOR-dependent upregulation of S6K2 allows S6K2 to interact with RORγ, hence accelerating the nuclear translocation of RORγ and Th17 differentiation. Additionally, the PI3K/Akt/mTORC1/S6K1 axis induces the downregulation of growth factor‐independent protein 1 (Gfi1), a negative regulator of Th17 differentiation in an S6K1/2-dependent manner (107).

**Figure 3:**
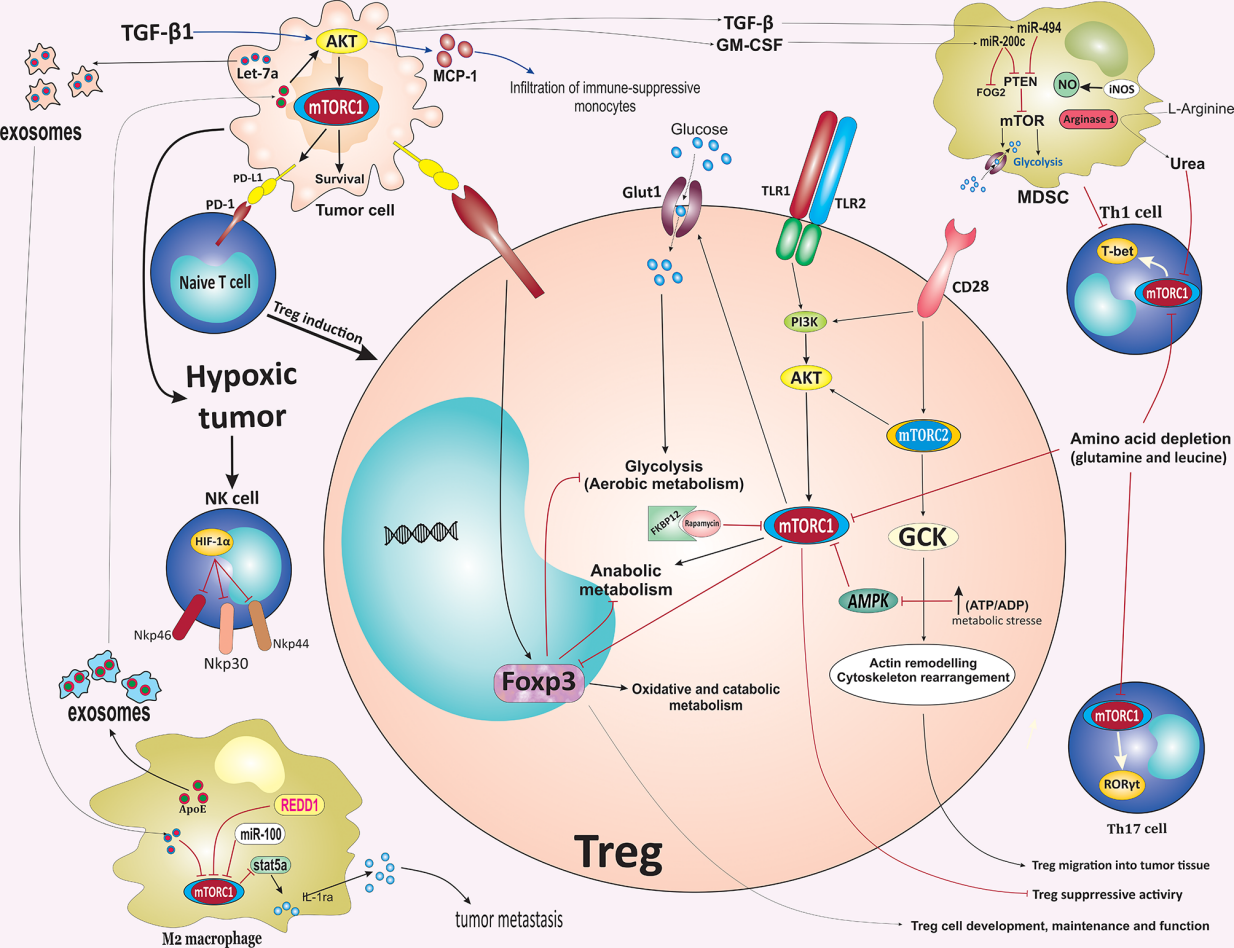
mTOR-mediated regulation of immune cells fate in the tumor microenvironment. Activation of mTORC2 following CD28 stimulation induces GCK expression. GCK promotes the migration of Tregs into tumor tissues *via* inducing rearrangement of the cytoskeleton. Inflammatory stimulus and Foxp3 reprogramming Treg cell metabolism by balancing mTORC1 signaling and glucose metabolism to promote the proliferation and suppressive function of Treg cells. TLR-mediated activation of mTOR impairs Treg cells suppressive capacity. In contrast to the mTOR pathway, Foxp3 reduces glucose uptake, glycolysis, and anabolic metabolism while enhancing oxidative and catabolic metabolism. AMPK activation following metabolic stresses such as increases in ATP: ADP ratio during hypoxia suppresses the expression of Glut1 and glycolysis in Tregs by modulating the mTORC1 signaling pathway, which reprograms Treg cell metabolism to promote mitochondrial oxidative metabolism rather than glycolysis. Interaction of PD-L1 on tumor cells with PD-1 on tumor-specific T cells promotes differentiation of naïve CD4^+^ T cells into Tregs. Downregulation of glutamine and leucine metabolism support Tregs differentiation while repressing the differentiation of Th1 and Th17 effector. In the TME, the upregulation of ARG-1 and iNOS expression in MDSCs results in the depletion of L-Arginine. ARG1-mediated hydrolysis of L-Arginine leads to urea production, which subsequently induces metabolic reprogramming of various cellular components in TME, especially T cells, by suppressing the mTOR signaling pathway. TAMs-derived exosomes enriched ApoE promotes the migration of gastric cancer cells by activating the PI3K/Akt/mTOR signaling pathway. Additionally, PI3K/ Akt/mTOR pathway increases the infiltration of immune-suppressive monocytes into tumor sites via inducing the expression of MCP-1 and IL-10, mainly through TGF-β1. Hypoxia-induced tumor exosomes that contain let-7a miRNA promote OXPHOS activity and downregulate insulin/Akt/mTOR signaling pathway in bone marrow-derived macrophages and promote polarization of infiltrating macrophages to an M2-like phenotype. mTOR inhibition suppressed the immunosuppressive function of MDSCs *via* blocking the iNOS pathway and ARG-1 activity. TAM, tumor microenvironment; GCK, glucokinase; mTOR, mammalian target of rapamycin; TLR, Toll-like receptor; Treg, regulatory T cell; Glut1, glucose transporter 1; ARG1, arginase 1; iNOS, inducible nitric oxide synthase; ApoE, apolipoprotein E; MCP-1, monocyte chemoattractant protein-1; ARG1, arginase 1; MDSC, myeloid-derived suppressor cells; PD-1, Programmed cell death protein 1; OXPHOS, oxidative phosphorylation; REDD1, regulated in development and DNA damage responses 1; TGF-β1, Transforming growth factor β1; Foxp3, factor forkhead box protein P3.

It has also been confirmed that the metabolic programming-mediated by mTOR in TME can influence the Treg differentiation, motility, and immunosuppressive functions. The migration of activated Treg cells to inflamed tissue, including TME, is critical for their immune-modulatory function. Migration of Treg cells requires glycolysis mediated by the enzyme glucokinase (GCK) induced by a PI3K-mTORC2 pathway. Activation of mTORC2, but not mTORC1, following CD28 stimulation on the surface of activated Treg, induces glucokinase expression. Subsequently, glucokinase induces the rearrangement of the cytoskeleton by triggering intense actin remodeling and ultimately promotes the migration of Tregs into tumor tissues **(** **Figure 3** **)** (108). The toll-like receptor (TLR)-mediated activation of the PI3K/Akt/mTORC1 signaling pathway also implicates metabolic reprogramming in Treg cells in TME that promotes Treg cell proliferation. It has been shown that ligation of TLR1 and TLR2 on activated Treg cells activates the PI3K/Akt/mTORC1 signaling pathway, which in turn promotes the expression of glucose transporter Glut1 on the cell membrane. Glut1 subsequently increases glucose uptake and therefore promotes glucose metabolism *via* glycolysis, supporting the energy demand for Treg cells proliferation and inflammatory function. However, TLR-mediated activation of the PI3K/Akt/mTORC1 signaling pathway impairs the immunosuppressive capacity of Treg cells. Conversely, the transcription factor Foxp3 opposed PI3K/Akt/mTORC1 signaling to reduce glucose uptake, glycolysis, anabolic metabolism while enhancing oxidative and catabolic metabolism. Hence, local inflammatory stimulus and Foxp3 reprogram Treg cell metabolism by balancing mTORC1 signaling and glucose metabolism to promote the proliferation and suppressive function of Treg cells (109). The above findings were consistent with some previous studies, for example, AMPK activation following metabolic stresses such as increased ATP: ADP ratios during hypoxia suppresses the expression of Glut1 and glycolysis in Tregs by modulating the mTORC1 signaling pathway and reprogramming Treg cell metabolism to promote mitochondrial oxidative metabolism rather than glycolysis **(** **Figure 3** **)** (110).

Interaction of PD-L1 on tumor cells with PD-1 on tumor-specific T cells promotes differentiation of naïve CD4^+^ T cells into Tregs, leading to immune suppression that supports tumor growth (111). Selective inhibition of mTORC1 signaling *via* deletion of RAPTOR reduces the PD-1, CTLA-4, and inducible T cell co-stimulator (ICOS) expression by effector Treg cells (112). It has also been revealed that PD-L1 expression regulates the development, maintenance, and functional properties of induced Treg (iTreg) through enhancing and sustaining the Foxp3 expression and hence, the suppressive function of iTreg cells (**Figure 3**) (111). Furthermore, both oncogenic and IFN-γ-mediated membranous expression of PD-L1 is associated with the Akt/mTOR pathway upregulation on NSCLC cells. Further study showed that the combination of mTOR inhibitor rapamycin and a PD-1 blocking antibody efficiently suppressed lung tumor growth (113).

Downregulation of glutamine and leucine metabolism repress the differentiation of Th1 and Th17 effector T cells while preserving Treg differentiation (96, 114, 115). The activation of the mTORC1 pathway is also essential for the expression of c-Myc, which plays a critical role in cell growth, differentiation, and various metabolic activities (116, 117). A decrease in the rates of glutamine metabolism led to the downregulation of the mTOR pathway and subsequently less Myc expression, thus resulting in a defect in the upregulation of the metabolic machinery essential for differentiation. In addition, glutaminase converts glutamine into glutamate to support the TCA cycle in growing cells (96, 114, 115). Glutamate deprivation or amino acid transporter SLC1A5 (ASCT2) deficiency was found to promote Treg cell generation (96, 118). In contrast to glutamine, the increased intercellular concentration of L-Arginine directly enhances remarkable metabolic reprogramming and survival capacity of CD4^+^ and CD8^+^ T cells, independently of mTOR signaling or downstream metabolites, thus, improves anti-tumor activity (119). Interestingly, elevated intracellular L-Arginine levels suppress T cell differentiation, improve T cell survival and sustain cells in a T central memory-like state. In the TME, the upregulation of ARG-1 and inducible nitric oxide synthase (iNOS) expression in MDSCs results in the depletion of L-arginine in the microenvironment which is necessary for T cell proliferation (120). ARG-1-mediated hydrolysis of L-arginine leads to the production of urea which subsequently induces metabolic reprogramming of various cellular components of local TME, especially T cells, by suppressing the mTOR pathway **(** **Figure 3** **)** (121). Hence, the beneficial influence of L-Arginine on T cell survival and anti-tumor functionality may be exploited therapeutically to enhance treatment regimens and better outcomes for cancer-bearing patients, for instance, to improve adoptive T cell therapies.

## mTOR Pathway Mediates Polarization of Tumor-Associated Macrophage Into M2 Macrophage

Macrophages are a highly heterogeneous population of immune cells and are distinctly subdivided into M1 and M2 phenotypes. Of these, M1 macrophages (classically activated macrophages), which develop in the presence of intracellular pathogens and their components, such as lipopolysaccharide (LPS) or Th1 cytokines (such as IFN-γ and TNF-α), are pro-inflammatory with cytotoxic properties and responsible for inflammatory signaling, while M2 macrophages (alternatively activated macrophages) that respond to type II cytokines such as IL-4 and IL-13 are anti-inflammatory macrophages that implicate in the restriction of the inflammatory process and prevent tumor cell attack by immune cells (122). Within the context of the TME, macrophages are the main and critical cell populations implicated in the inflammatory process associated with tumor growth and progression (123). TAMs mainly represent an M2-like phenotype, the major population of infiltrating inflammatory immune cell components of the TME, and play a key role in promoting tumorigenesis through mechanisms such as stimulation of angiogenesis, enhancement of tumor migration, immune evasion, chemoresistance, and exert local immunosuppressive effects (124). Metabolic reprogramming-mediated by Akt/mTOR pathway has been shown to be required for determining the activation status of macrophages, the polarization of macrophages toward the M1 or M2 phenotype, and the acquisition of macrophages effector activity, depending on the context in which they are, including the TME (125). Pharmacological inhibition of mTOR by rapamycin has been found to affect macrophage survival and polarization by inducing macrophage apoptosis during M0/M2 and reducing M2 polarization and conversely promoting a shift to an M1-like profile and enhancing M1 phenotype polarization in human and murine macrophages. Beyond the impact on macrophage polarization, mTOR inhibition was associated with modification on macrophage phenotype and cytokine/chemokine secretion profile, with the M2 most profoundly affected (126, 127). Accumulation of TAMs associate with tumor progression and angiogenesis. mTOR pathway is crucial element in the regulation of monocyte polarization into TAMs. Rapamycin treatment promotes the differentiation of monocytes into M1 macrophages releasing more IL-12 and less IL-10, whereas TSC2 knockdown-mediated mTOR activation caused the monocytes to differentiate into M2 macrophages releasing less IL-12 and more IL-10. Furthermore, infusion of mice with TSC2-deficient or TSC2-overexpressing monocytes promote or reduces tumor angiogenesis and growth in murine xenografts by modulating macrophage polarization, respectively (128). Zhao et al. demonstrated that renal cell carcinoma cells are able to recruit macrophages into TME through increasing C-X-C motif chemokine ligand 8 (CXCL8) cytokine expression. Increased infiltrating macrophages were associated with increased renal cell carcinoma cells invasion capabilities and metastasis *via* inducing the epithelial-mesenchymal transition (EMT) and increased cancer stem cell-like populations by activating the Akt and mTOR signaling pathway (129). In addition, TAMs-derived exosomes enriched apolipoprotein E (ApoE) promote the migration of gastric cancer cells by activation of PI3K/Akt/mTOR signaling pathway **(** **Figure 3** **)** (130).

M1 and M2 macrophages require distinct metabolic programs. While M1 macrophages are known to rely on aerobic glycolysis and lipogenesis programs, M2 macrophages increase glucose utilization, upregulate fatty acid oxidation (FAO), and oxidative phosphorylation (OXPHOS) (131, 132). Macrophage colony-stimulating factor (M-CSF) synergizes with IL-4 couple mTORC2 activation with STAT6 signaling which enhances glycolysis during M2 macrophage activation *via* the induction of the transcription factor interferon regulatory factor 4 (IRF4) (132). Additionally, PI3K/Akt signaling axis was shown to increase the infiltration of immune-suppressive monocytes to tumors *via* monocyte chemoattractant protein-1 (MCP-1) expression and IL-10, mainly through TGF-β1 **(** **Figure 3** **)** (133).

Immunosuppressive TME contains a large number of arginine1 positive (ARG1^+^) macrophages (121, 134). ARG1 production by macrophages stimulated *via* Th2 cytokines, myeloid suppressor cells, and peripheral myeloid cells in the TME downregulates CD3ζ expression, a hallmark of T cell dysfunction in cancer patients, which results in suppression of TCR expression and antigen-specific T cell response (135–137). Additionally, arginine-starvation arrests T cells in the G0/G1 phase of the cell cycle as a result of an impaired expression of cyclin D3 and cyclin-dependent kinase 4 (cdk4) in T cells by reducing mRNA stability and ultimately downregulation of translational rate (138, 139). These results may help develop novel immunotherapeutic avenues to target arginase as an important step in the success of immunotherapy. TAMs-derived products can mediate the activation of PI3K/Akt/mTOR, which, in turn, is associated with drug resistance cancer.For example, enhanced CC‐chemokine ligand 2 (CCL2) secretionby TAMs increases endocrine resistance in breast cancer cells via activation of the PI3K/Akt/mTOR signaling pathway. Reciprocally, endocrine‐resistant breast cancer cells activate the mTORC1‐FOXK1 pathway of macrophages by altering amino acid metabolism in the microenvironment, which enhances M2 macrophage polarization and CCL2 secretion by macrophages. Thus, CCL2 plays a critical role in this malignant feedback loop. Additionally, a high expression level of CCL2 in the stroma is associated with infiltration of CD163^+^ macrophages and poor progression‐free survival (PFS) of patients with estrogen receptor‐positive breast cancer. CD163 is a member of the scavenger receptor cysteine-rich (SRCR) protein family, which is considered a highly specific monocyte/macrophage marker for polarization of M2‐type macrophages (140). Interestingly, IL-4 signaling co-opts the Akt-mTOR signaling axis to induce the expression of a subset of M2-associated genes by promoting the glucose uptake, glycolysis, and production of cytosolic acetyl-CoA. An increased acetyl-CoA activity mediates epigenetic reprogramming through gene-specific histone acetylation to control M2 polarization and activation (141).

Hypoxia conditions have been revealed to promote tumor secretion of exosomes enriched in immunosuppressive components. Hypoxia-induced tumor exosomes containing let-7a microRNA (miRNA) enhance mitochondrial OXPHOS activity and suppress insulin/Akt/mTOR signaling pathway in bone marrow-derived macrophages (BMMs), as a result, promotes polarization of infiltrating macrophages to an M2-like phenotype. Therefore, biomolecule-loaded exosomes, such as let-7a, enhance tumor immune evasion and tumor progression by promoting changes in immunometabolic profile of infiltrating monocyte-macrophage population **(** **Figure 3** **)** (2, 142). miR-100 is highly expressed in TAMs and promotes M2-polarization of macrophages, and maintains TAMs phenotype through the downregulation of the mTOR signaling pathway. Furthermore, elevated expression of miR-100 in TAMs is associated with secretion of immunosuppressive cytokine IL-1ra through stat5a-mediated transcriptional regulation, which promotes cell stemness and tumor metastasis via stimulating the Hedgehog signaling pathway. These findings highlight that the mTOR pathway/miR-100/IL-1ra axis may play an essential role in maintaining the TAMs phenotype and promoting tumor metastasis **(** **Figure 3** **)** (143).

miR-484 exhibits significant anticancer properties by indirectly affecting mTOR-mediated macrophage activation *via* inhibition of CD137L. CD137L promotes cell viability *via* the PI3K and mTOR cell pathways and increases IL-8 (CXCL8) production, which results in promoted recruitment of macrophages and neutrophils into the TME (144).

Hypoxic TAMs acquire metabolic changes that promote their angiogenic and immunosuppressive properties. REDD1, a negative regulator of mTOR significantly upregulates in hypoxic TAMs results in suppressing glycolysis in TAMs and inhibiting their excessive angiogenic response. REDD1 deletion in TAMs increases glucose uptake by upregulating Glut1 and glycolysis in an mTOR-dependent manner. These events enhance their rates of glycolysis to a level that competes with neighbouring endothelial cells for glucose, resulting in reducing endothelial glucose availability and persuading them for competition with TAMs for glucose uptake. This metabolic competition over glucose promotes the formation of organized tumor vasculature restores oxygenation within the tumor, and prevents metastases. These results exhibit the functional link between TAM metabolism in hypoxia and tumor angiogenesis (145).

In this regard, targeting the mTOR pathway can be used as a potential therapeutic strategy in an attempt to modulate macrophage responses in the context of TME to promote antitumor immunity with therapeutic benefit in a broad range of cancers.

## mTOR Pathway Drives Tumor-Induced Myeloid-Derived Suppressor Cells Accumulation to Induce Tumor Progression

MDSCs represent a population of a heterogeneous group of immature myeloid cells defined as CD11b^+^ Gr1^+^ cells that are characterized by the ability to suppress both adaptive and innate immunities during cancer, infection, and inflammatory diseases. Based on Ly6G and Ly6C expression, MDSCs can be further stratified into two distinct subsets of CD11b^+^ Ly6G^+^ Ly6C^low^ granulocytic MDSC (G-MDSC) and CD11b^+^ Ly6G^+^ Ly6C^high^ monocytic MDSC (M-MDSC) cells. MDSCs are known as ‘queen bee’ of TME as they can suppress both innate and adaptive immunity through diverse mechanisms, especially to suppress T cell responses (146, 147). Recently, mTORC1 has been demonstrated to play a pivotal role in the differentiation and function of MDSCs. Specific inhibition of mTORC1 signaling by either immunosuppressant drug rapamycin or genetic deletion decreased the differentiation and accumulation of M-MDSCs in tumors and skin allografts. Alternatively, in conditional disruption of mTORC2 signaling *via* deletion of RICTOR, the differentiation of MDSCs remained unaffected. Furthermore, mTOR inhibition suppressed the immunosuppressive function of MDSCs *via* blocking the iNOS pathway and ARG-1 activity. Additionally, mTORC1 activity is necessary for fine-tuning the immunosuppressive CD11b^+^ Ly6C^high^ M-MDSC maturation and their functional properties by enhancing cellular glycolysis activity **(** **Figure 3** **)** (146). Tumor-infiltrating M-MDSCs represent a high level of mTOR phosphorylation. In turn, mTOR-mediated glycolysis is associated with the promoted suppressive function of tumor-infiltrating M-MDSCs (146). In the TME, the upregulation of ARG-1 and iNOS expression in MDSCs leads to the depletion of L-Arginine in the microenvironment, which is necessary for T cell proliferation (120). Further studies for identification of the role of mTOR in the differentiation of MDSCs showed that pharmacological inhibition of mTOR by rapamycin significantly reduces glucose uptake and lactate production during MDSCs induction. Rapamycin treatment reduces the expression of genes encoding glycolytic enzymes, including the transporter Glut1 and glycolytic enzymes hexokinase 2 (HK2), phosphofructokinase 1 (PFK1), pyruvate kinase muscle (PKM), and LDHA (lactate dehydrogenase-α) in CD11b^+^Ly6C^high^ M-MDSCs **(** **Figure 3** **)** (148). mTOR-mediated upregulation of granulocyte colony-stimulating factor (G-CSF) in tumor-initiating cells (TICs) dictates the recruitment of pro-tumorigenic MDSCs into TME. Accumulated MDSCs reciprocally increase TICs frequency by upregulating Notch signaling within tumor cells, suggesting a potential role of mTOR–G-CSF axis in creating a feed-forward loop between TICs and within the TIME. Furthermore, rapamycin-mediated inhibition of mTOR leads to reduced TIC levels (149). TGF-β is another factor that promotes the recruitment of MDSCs to the TME. TGF-β directly induces differentiation of MDSCs into CD39^high^/CD73^high^ pro-tumorigenic terminally differentiated myeloid mononuclear cells (TDMMCs) (150). CD39^+^CD73^+^ MDSCs have been reported to be dominantly accumulated in the tumor and peri-tumoral stroma of NSCLC patients which is characterized by enriched suppressive molecular signatures. TGF-β-mediated upregulation of mTOR resultes in the activation of hypoxia-inducible factor-1α (HIF-1α) that induces CD39/CD73 expression on tumor-infiltrating MDSCs. Indeed, rapamycin-mediated inhibition of mTOR abrogates the TGF-β-mediated induction of CD39/CD73 expression on MDSCs by disruption of HIF-1α (151). Additionally, tumor-infiltrated MDSCs can dictate their tumor-promoting effect by determining the fate of the TME components. It has been shown that conditioning of T cells with MDSCs leads to reduced mTOR activity in T cells as well as increased adoptive T cell-based immunotherapy (ACT) anti-tumor efficacy, suggesting the critical role of MDSCs in mTOR-mediated CD8^+^ T cells differentiation into effector populations (152). However, several lines of evidence have identified that the mTOR inhibition promotes the development of memory CD8^+^ T cells (13, 77, 81).

Tumor-derived factors also play an indispensable role in the upregulation of specific miRNAs in MDSCs to regulate molecular networks controlling the accumulation and function of tumor-infiltrating MDSCs. TGF-β1-mediated upregulation of miR-494 in MDSCs supports tumor cells proliferation and metastasis by regulating the activity of MDSCs *via* targeting mTOR inhibitor PTEN. Similarly, tumor-derived granulocyte‐macrophage colony‐stimulating factor (GM‐CSF) induces the expression of miR-200c in tumor environment, and miR-200c, in turn, enhances the expansion and immune suppressive potential activity of MDSCs through negatively targeting PTEN and friend of Gata 2 (FOG2) expression. FOG2 and PTEN downregulation lead to activation of the PI3K/Akt/mTOR signaling pathway to enhance the expansion and immune suppressive potential of MDSCs **(** **Figure 3** **)** (153). Thus, tumor-derived factors induced by miRNAs support the tumor growth and progression by regulating the activity of MDSCs *via* targeting PTEN, which activates the Akt/mTOR signaling axis.

Taken together, these findings highlight the potential role of mTOR signaling in the differentiation and recruiting pro-tumorigenic MDSCs cells to dictate an immunosuppressive tumor environment.

## mTOR-Dependent Mechanisms in Tumor Microenvironments Polarize Neutrophils Toward Pro-Tumoral Phenotypes

Neutrophil granulocytes have long been regarded as the most abundant type of granulocytes and the first line of defence against infections and inflammatory conditions. Whereas, several reports have provided evidence for tumor-associated neutrophils (TANs) with potential anti-tumor and anti-metastatic properties (N1 phenotype) with direct cytotoxicity toward tumor cells, others have reported their pro-tumor functions (N2 phenotype) such as supporting angiogenesis, degrading extracellular matrix, promoting the migratory and invasive potential of tumor cells, and shaping the TME toward a more immunosuppressive state (154). Both mTORC1 and mTORC2 complexes are remarkably implicated in the regulation of numerous neutrophil functions, such as chemotaxis, neutrophil extracellular trap (NET) formation, and expression of pro-inflammatory cytokines (12). mTORC2-dependent regulation of Myosin II is essential for neutrophil polarity and migration (155). It has been exhibited that TME resident immune cell subpopulations such as TAMs and TANs secret soluble factors such as Oncostatin M (OSM), an inflammatory cytokine belonging to the IL-6 superfamily, which in turn, promotes polarization of TAMs into pro-tumorigenic M2 phenotype in a mTORC2/Akt1-dependent manner **(** **Figure 2** **)** (156). However, hepatoma-derived soluble factors, including hyaluronan fragments, upregulate the functional LC3 and autophagosomes in neutrophils, resulting in increased autophagy which was unrelated to the deactivation of the mTOR signaling pathway. Interestingly, upregulated neutrophil autophagy was associated with prologue production of pro-metastatic OSM and MMP-9 and promoted metastasis (157). Senescence-associated secretory phenotype (SASP) process refers to the accumulation of proinflammatory mediators and growth-promoting factors secreted by senescent cells in the NF-kB and STAT3 signaling-dependent manner. Loss-of-function mutations of oncogenic molecules such as P53, RAS, Notch or mTOR pathway cause to an alternative SASP that correlated with the induction of chronic inflammatory conditions providing an immunosuppressive TME. In this context, immunosuppressive myeloid cells, including macrophages and neutrophils, suppress NK and CD8^+^ T cell-mediated anti-tumor response and hence augment tumorigenesis (158). The mTOR-S6K1 pathway plays a key role in the neutrophil chemotaxis. In the presence of either GM‐CSF or IL‐8, pre‐incubated neutrophils with rapamycin significantly inhibits neutrophil chemotaxis and chemokinesis. Rapamycin also inhibits GM‐CSF‐induced enzymatic activity and actin polymerization, a hallmark of leukocyte migration (159).

These findings highlight the critical role of the mTOR pathway in migratory potential and polarization of TANs toward pro-tumoral phenotypes.

## mTOR-Mediated Regulation of Dendritic Cells in Tumor Microenvironment

DCs have a wide range of antigen presentations and are essential for the activation of both helper CD4^+^ and cytotoxic CD8^+^ T cells, especially for processing tumor antigens and priming anti-tumor immunity. The PI3K/Akt/mTOR constitutes a critical pathway downstream of the cytokine FMS-like tyrosine kinase 3 ligand (Flt3L) in is important for DCs subsets development and function, particularly for plasmacytoid DCs (pDCs) and CD8^+^ DCs (13). A more recent study showed that treatment of mature bone marrow monocyte (BMM)-derived DCs with various inhibitors of mTOR (mTORi) promote their antigen-presenting and processing abilities, as well as these cells tended to be non-apoptotic by reducing the expression of apoptotic molecules. In addition, the cytotoxic CD8^+^ T lymphocytes-mediated killing of tumor cells increases following activation of mTORi-treated BMM-derived DCs. Interestingly, *in vivo* study demonstrated that the mice-bearing tumor treated with both mTORi and connective tissue growth factor (CTGF)/E7 DNA vaccine had higher percentages of mature DCs in the TME with better disease control and prolonged survival. These results revealed that utilization of mTOR inhibitor could be a potential pharmacological approach for temporally extending life span, antigen-presenting and antigen processing of DCs to improve the therapeutic outcome of cancer immunotherapy (160). Inter-tumoral delivery of mTORC2-deficient DCs (Rictor−/− DCs) showed pro-inflammatory properties and was associated with reduced melanoma tumor growth, increased numbers of INF-γ+ and granzyme B+ (GrB+) CD8^+^ TILs, and reduced frequency of immunosuppressive MDSCs within TME (161). These findings raise the possibility that therapeutic inhibition of mTORC2 may present an effective strategy to enhance the therapeutic efficacy of DC-based vaccines for cancer immunotherapy. PI3K/Akt/mTOR signaling axis is critically implicated in regulating the metabolic demands essential for DC activation. It has been shown that TLR-driven mTOR signaling negatively regulates costimulatory molecule expression following LPS stimulation of DCs. Indeed, inhibition of mTOR activity through rapamycin augments the expression of costimulatory molecules, extends the life span, and prolonged activation kinetics of activated DC, *via* induction of glycolytic metabolism. Furthermore, rapamycin treatment improves the capacity of DCs to augment the induction of primary antigen-specific CD8^+^ T cell responses and induces effective anti-tumor responses by inducing efficient and sustained CTL responses in a therapeutic vaccination treatment model (162). Suggesting that mTOR signaling in DCs is involved in the establishment of an immunosuppressive environment.

## mTOR Pathway Regulates NK and NKT Cells Metabolic Activity and Proliferation

NK cells are known to play an essential role in cancer control. IL-15 stimulation promotes NK cell metabolism *via* prompting intracellular mTOR signaling which, is vital for sustaining the NK cells proliferation, metabolism, and for achieving antitumor cell lysis (163). Conversely, TGF-β-mediated inhibition of mTOR signaling can directly suppress the metabolism and activation of NK cells (164), suggesting an mTOR-dependent immune suppressive role for TGF-β in TME. In NK cells, the mTOR pathway is essential for metabolic response by upregulating glucose uptake and glycolysis (163). HIF-1α expression depends on the mTOR signaling pathway. Hypoxic environments promote the overexpression of HIF-1α which causes the downregulation of NK activating receptors NKp46, NKp30, NKp44, and NKG2D (165). Additionally, low-arginine or glutamine conditions significantly suppress mTOR signaling within NK cells, which affect c-Myc expression in IL-2/IL-12-stimulated NK cells **(** **Figure 3** **)** (166). Interestingly, gastric cancer mesenchymal stem cells (GCMSCs) impair NK cell function through mTOR signaling (167). In addition to the key roles of mTORC1 for NK cell responses, it is worth noting that continuous exposure of NK cells with IL-15, which is essential for stimulating mTORC1 signaling, drives NK cell exhaustion and reduced cytotoxicity (168).

NKT cells are subsets of T lymphocytes and act as a bridge between innate and adaptive immunity. In addition to killing the CD1d expressing tumor cells directly, activated NKT cells are able to promote the killing effects of NK cells and CTLs against tumor cells (169). Lactic acid production and secretion following Warburg glycolysis by cancer cells results in acidification of the TME, suppresses PI3K/Akt/mTOR pathway, and therefore inhibits T cell glycolysis (170, 171). However, the influences of TME on NKT cells functions remain to be fully elucidated. It has been shown that the accumulation of lactic acid in TME suppresses IL-4 and especially diminishes IFN-γ productions by NKT cells. NKT cell dysfunctions were restored upon adjusting PH to neutral values. The further experiment demonstrated that extracellular acidification inhibited NKT cell functions by inhibiting mTOR signaling and nuclear translocation of promyelocytic leukemia zinc finger (PLZF) (172), a critical transcription factor for NKT cells development and effector function (173). mTOR-mediated regulation of PLZF plays a crucial in NKT cell lineage development and effector function (173).

## Conclusion

As was mentioned above, it is well established that upregulation of the PI3K/Akt/mTOR network is critical in promoting tumor pathogenesis by shaping the characterization and the activity of the TME’s elements specially recruited immune cells. Although inhibition of mTOR promotes cancer cell death by promoting apoptosis and creating nutrient deprivation conditions in cancer cells, mTORC1 blockade can efficiently enhance prolonged protective immunity by stimulating the generation of long-lived protective memory T cells. In addition, utilization of mTOR inhibitors can also affect the fates of other immune cells recruited to TME such as CD4^+^ and CD8^+^ T effector cells, Th17, Tregs, and macrophages, all of which appear to utilize this crucial metabolic regulator for their differentiation and function. Improving immune responses by manipulating cellular metabolic pathways in combination with other anti-cancer agents may provide new options for cancer immunotherapy. In general, metabolite levels can be affected in the absence of genetic manipulations, suggesting the promising opportunity and challenge for therapeutic applications. Additionally, our knowledge of the dynamics of the proteome and metabolome during the immune response constitutes a framework for further studies addressing the complex interplay between metabolism and cellular functions. Concerning TME biology and its potential role as a therapeutic target, the balance between different T cell subsets and recruitment and activation of immunosuppressive myeloid subsets can be altered by multiple tumor-derived factors released in the context of TME, resulting in mTOR-mediated metabolic and transcriptional reprogramming and the establishment of inappropriate immune cell responses. However, further studies are required to examine how extracellular signals affect mTOR in regulating immune responses in TME. Hence, combining mTOR inhibitors and cell-based immunotherapies in cancer treatment could be therapeutic strategies to increase the antitumor efficacy of immunotherapies. As a consequence, direct manipulation of immune cells metabolism by manipulating the PI3k/Akt/mTOR axis has the potential to provide a new avenue for cancer immunotherapy.

## Author Contributions

SM, conceptualization, writing‐original draft, and figure design. BM, conceptualization, figure design, writing‐review and editing. HS, writing‐review and editing, and visualization. RA and ST, writing‐original draft and editing. WC, project administration, writing‐original draft, writing‐review and editing. DR, project administration, writing‐original draft, figure design, and writing‐review and editing. All authors contributed to the article and approved the submitted version.

## Funding

This review is supported by a grant from the Medicinal plant research, Yasuj University of Medical Sciences, Yasuj, Iran.

## Conflict of Interest

The authors declare that the research was conducted in the absence of any commercial or financial relationships that could be construed as a potential conflict of interest.

## Publisher’s Note

All claims expressed in this article are solely those of the authors and do not necessarily represent those of their affiliated organizations, or those of the publisher, the editors and the reviewers. Any product that may be evaluated in this article, or claim that may be made by its manufacturer, is not guaranteed or endorsed by the publisher.
